# Proteomic profiling of the thrombin-activated canine platelet secretome (CAPS)

**DOI:** 10.1371/journal.pone.0224891

**Published:** 2019-11-13

**Authors:** Signe E. Cremer, James L. Catalfamo, Robert Goggs, Stefan E. Seemann, Annemarie T. Kristensen, Marjory B. Brooks

**Affiliations:** 1 University of Copenhagen, Department of Veterinary Clinical Sciences, Copenhagen, Denmark; 2 Cornell University, Department of Population Medicine and Diagnostic Sciences, Ithaca, New York, United States of America; 3 Cornell University, Department Clinical Sciences, Ithaca, New York, United States of America; 4 University of Copenhagen, Department of Veterinary and Animal Sciences, Copenhagen, Denmark; Institut d'Investigacions Biomediques de Barcelona, SPAIN

## Abstract

Domestic dogs share the same environment as humans, and they represent a valuable animal model to study naturally-occurring human disease. Platelet proteomics holds promise for the discovery of biomarkers that capture the contribution of platelets to the pathophysiology of many disease states, however, canine platelet proteomic studies are lacking. Our study objectives were to establish a protocol for proteomic identification and quantification of the thrombin-activated canine platelet secretome (CAPS), and to compare the CAPS proteins to human and murine platelet proteomic data. Washed platelets were isolated from healthy dogs, and stimulated with saline (control) or gamma-thrombin (releasate). Proteins were separated by SDS-page, trypsin-digested and analyzed by liquid chromatography and tandem mass spectrometry (MS). CAPS proteins were defined as those with a MS1-abundance ratio of two or more for releasate vs. unstimulated saline control. A total of 1,918 proteins were identified, with 908 proteins common to all dogs and 693 characterized as CAPS proteins. CAPS proteins were similar to human and murine platelet secretomes and were highly represented in hemostatic pathways. Differences unique to CAPS included replacement of platelet factor 4 with other cleavage products of platelet basic protein (e.g. interleukin-8), novel proteins (e.g. C-C motif chemokine 14), and proteins in relatively high (e.g. protease nexin-1) or low (e.g. von Willebrand factor) abundance. This study establishes the first in-depth platelet releasate proteome from healthy dogs with a reference database of 693 CAPS proteins. Similarities between CAPS and the human secretome confirm the utility of dogs as translational models of human disease, but we also identify differences unique to canine platelets. Our findings provide a resource for further investigations into disease-related CAPS profiles, and for comparative pathway analyses of platelet activation among species.

## Introduction

Comparative studies provide insight into platelet structure, function, and signaling pathways. For example, species differences in the platelet open canalicular system have been exploited to study the kinetics of granule secretion and platelet spreading [1]. Domestic dogs have similar platelet counts and genetic diversity as human beings, and uniquely among model systems, companion dogs share the same environment as their owners. The dog is an important large animal model that has features that complement rodent models used for biomedical research. Several disease conditions with either primary or secondary platelet involvement, e.g. inherited platelet defects (such as Glanzmann thrombasthenia [2–4], Scott Syndrome [5, 6], dense granule storage pool disease [7]), von Willebrand disease [8], disseminated intravascular coagulation [9], immune mediated thrombocytopenia [10], and cancer [11, 12], are shared by dogs and humans. Dogs with single-gene defects that parallel human hereditary disease have moreover been used to develop and evaluate the efficacy of novel treatments, including platelet-targeted gene therapy [13]. The application of animal model systems however, has proven more challenging when studying the role of platelets in complex inflammatory, neoplastic and degenerative disease syndromes.

In human medicine, platelet proteomics have been used to identify novel proteins and to characterize changes in proteins in response to activation stimuli or associated with various disease states [14]. Sample-types for these analyses have included resting platelet lysates [15], isolated granules [16–19], releasates [20–30], membrane fractions [31, 32] and microparticles [33–37]. Proteomic analyses of platelets from patients with coronary artery disease have been used to search for clinically relevant disease biomarkers and novel drug targets [38–41]. A proteomic approach has also been applied to define the contribution of platelets to the pathogenesis of sepsis [42], Alzheimer’s disease [43], diabetes mellitus [44], and uremia [45]. Common to these clinical studies are the altered platelet protein profiles and the emerging evidence that platelet-derived proteins might provide disease-specific biomarker fingerprints. With the exception of a single study of hereditary canine Scott syndrome, in which proteomics confirmed a mutation of a platelet membrane protein as the causative platelet defect [46], no platelet proteomic investigations have been undertaken in dogs.

To extend the utility of dogs as a model system for inherited and acquired disease, and to provide a healthy CAPS reference profile for future disease-related canine studies, the present study aimed to establish a protocol for the identification and quantification of proteins secreted from activated canine platelets. The protein signature of the gamma-thrombin induced canine platelet secretome (CAPS) was established in healthy dogs using shotgun proteomics. We compared all identified canine platelet proteins with a previously described global human platelet proteome [15], and compared CAPS proteins with human [29] and murine [47] platelet secretomes. As shotgun proteomic profiling has never been reported in dogs before, the established protocol was based on the human literature, with necessary adjustments implemented to accommodate for species differences, as detailed below.

## Materials and methods

### Blood sampling and platelet isolation

**Fig 1:**
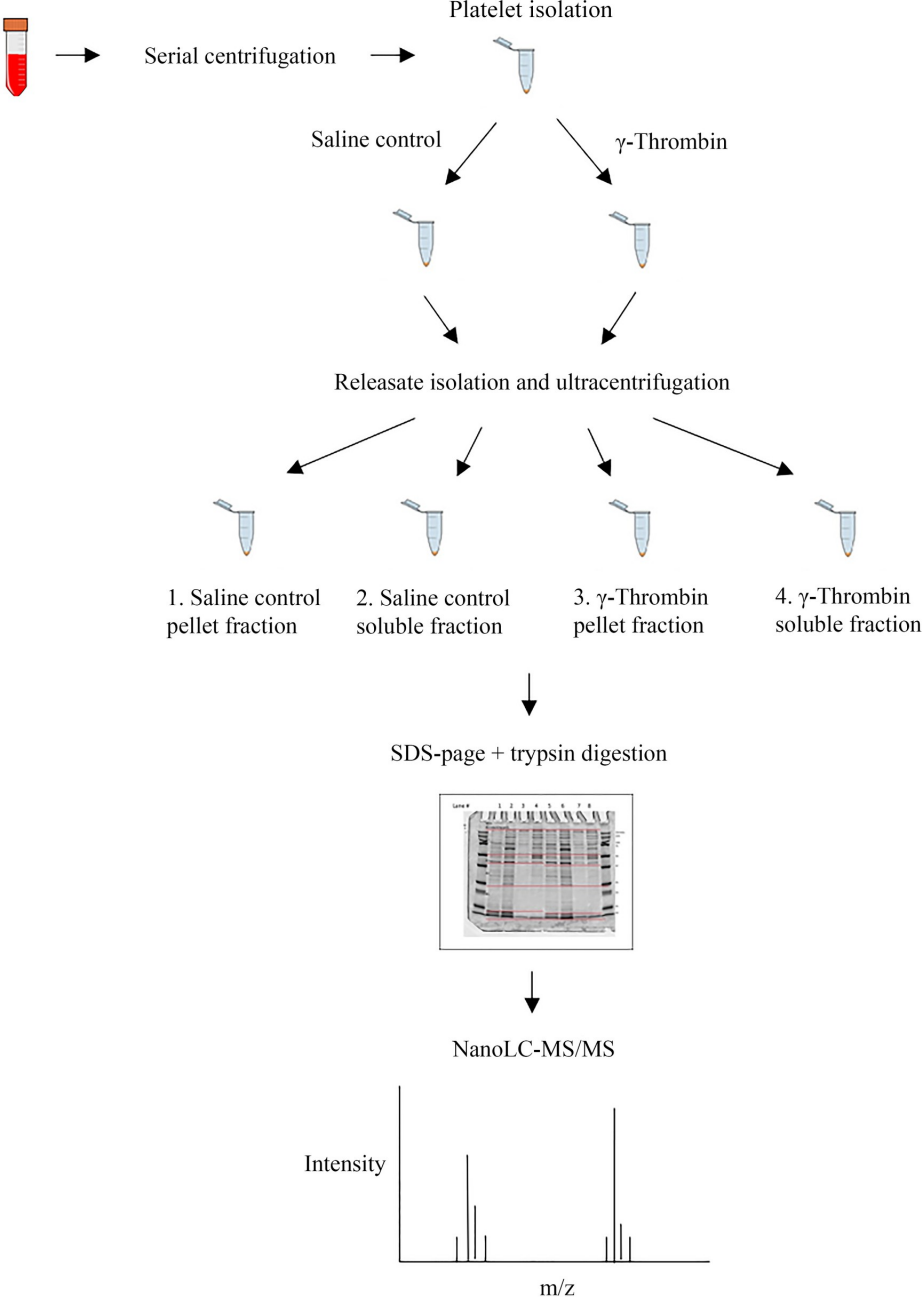
Experimental design flow chart. Platelets were isolated and washed free of plasma by serial centrifugation, divided into two aliquots and activated with saline (control) or gamma-thrombin. The releasate was cleared of debris and then fractionated by ultra-centrifugation into particulate (pellet) and soluble fractions. Proteins were separated by SDS-page, trypsin digested and analyzed by nanoLC-MS/MS.

The work flow is depicted in Fig 1. Acid citrate dextrose (ACD-A) anticoagulated whole blood samples (18 mL) were obtained from the cephalic vein of three healthy dogs as previously detailed [5]. One dog was a client-owned 5-year-old mixed breed spayed female and the other two dogs were a 6-year-old castrated male hound and a 3-year-old intact male beagle housed at an American Association for Accreditation of Laboratory Animal Care-approved facility. All procedures for animal use were reviewed and approved by the Institutional Animal Care and Use Committee at Cornell University (IACUC protocol no. 1994–0089), and owner consent was obtained. To account for the smaller body size and blood volume of dogs, the sample volume collected was less than used in human studies [24, 25, 29]. In order to maximize the platelet yield from canine whole blood, the samples were diluted to a final volume of 50 mL with wash buffer (113 mM NaCl, 4.3 mM K_2_HPO_4_, 4.2 mM Na_2_HPO_4_, 24.4 mM NaH_2_PO_4_, 5.5 mM glucose, pH 6.3) plus 1.25 μM PGE_1_ (P7527, Sigma Aldrich) and 0.04 U/mL apyrase (Sigma type VII, A6535, 200 U/mg protein). The samples were gently mixed and then centrifuged in a swing-out rotor (Sorvall Legend XTR Centrifuge, ThermoFisher Scientific US) at 650 x *g* for 5 min at room temperature (RT) without braking (remaining centrifugations were performed with brake). Thirty milliliters of the resultant dilute platelet rich plasma (dPRP) supernatant was collected, and to further maximize the platelet yield, the remaining 20 mL resuspended in 30 mL wash buffer plus 0.04 U/mL apyrase. The resuspended cells were centrifuged at 650 x *g* for 5 min at RT. Platelets in the upper 30 mL of this second supernatant were removed and combined with the first 30 mL dPRP, with the addition of wash buffer with 0.04 U/mL apyrase to a final volume of 100 mL. The dPRP (60 mL total) was mixed, and a 500 μL aliquot was removed for a complete blood cell count (CBC) (Advia2120 Hematology System, Siemens, USA). Equal volumes of dPRP were divided between two 50 mL polypropylene tubes and spun at 1,250 x *g* for 1 min at RT, to remove contaminating RBCs and WBCs. The supernatant was divided equally between four 50 mL polypropylene tubes, and the platelets sedimented at 1,250 x *g* for 10 min at RT to achieve efficient washed platelet recovery. The supernatant was discarded, and the inside of the tube wiped dry to further remove plasma proteins. The platelets were subjected to a final wash step by resuspending the four platelet pellets in 80 mL of wash buffer containing 0.02 U/mL apyrase. Each wash step was followed by collection of a 500 μL aliquot for a CBC. The platelets were pelleted by centrifugation at 1,250 x *g* for 10 min at RT. The supernatant was removed, the inside of the tubes wiped dry once more for removal of contaminants, and the washed platelets resuspended in 2.1 mL activation buffer (137 mM NaCl, 4 mM KCl, 0.5 mM MgCl-6H_2_O, 0.5 mM Na_2_HPO_4_, 0.1% glucose, 30 mM HEPES, pH 7.4). An aliquot of 25 μL was removed into 475 μL wash buffer for a CBC. The number of recovered washed platelets was 1.1 x 10^9^/mL, 5.8 x 10^8^/mL and 4.0 x 10^8^/mL, respectively, corresponding to concentrations reported in human studies [20, 26, 30, 48–50]. Washed platelet concentration was not adjusted to a standardized number because platelet yield varied; dilution would have resulted in washed PRP concentrations below acceptable limits for activation. Prior to activation, the platelets were rested for 10 minutes at 37°C followed by 20 minutes at RT for platelet cAMP levels to return to basal levels.

### Baseline ex-vivo platelet activation

Flow cytometry was utilized to detect *ex-vivo* platelet activation due to washing, evaluate the response of washed platelets to agonist stimulation, and to detect residual leukocytes in the platelet suspensions. Two microliters of post-washing samples were diluted in 1 mL PBS containing 0.4 mM gly-pro-arg-pro-NH_2_ acetate (Sigma Aldrich, USA) and stimulated with human gamma-thrombin (Haematologic Technologies Inc., USA) at 50 nM final concentration for 10 min at 37°C without stirring. Platelets were labelled with mouse monoclonal anti-human CD9-FITC (clone H19a, Biolegend, San Diego, CA, USA, Antibody Registry Identifier: AB_314908) [51] in a 1:1000 final dilution and either mouse monoclonal anti-human CD62P-PE (clone AC1.2, BD Biosciences, San Diego, CA, USA, Antibody Registry Identifier: AB_2184974) [52] in a 1:10 final dilution to detect P-selectin expression or rat monoclonal anti-human CD18-PE (Clone YFC118.3, AbD Serotec, Oxford, England, Antibody Registry Identifier: AB_321328) in a 1:20 final dilution to identify leukocytes [51]. An isotype antibody conjugated with PE (BD, Biosciences) was used to define nonspecific antibody binding. Cell suspensions were incubated in the dark for 20 min at RT, quenched with 500 μL PBS and analyzed within 30 min on a flow cytometer (FacsCalibur, BD Biosciences, USA). Platelet populations were defined based on forward scatter (FCS), side scatter (SSC) and CD9 fluorescence. Degranulation, based on CD62P-PE fluorescence within the platelet gate, was determined with and without thrombin activation (S1 Fig). Leukocyte contamination was detected based on the presence of CD18 positive events counted within predefined leukocyte gates (S2 Fig).

### Gamma-thrombin activation and releasate collection

Platelet aggregation was monitored using a PAP-8E light-transmission aggregometer (Bio/Data Corporation, USA). Briefly, 450 μL of rested, washed platelets suspension and 4.5 μL 200 mM CaCl_2_ were added to 4 individual cuvettes and stirred at 1,000 RPM for 2 min at 37°C prior to addition of 50 μL vehicle control (0.15 M NaCl) or gamma-thrombin (50 nM final concentration). The reaction proceeded with continuous recording for control and gamma-thrombin stimulated samples, respectively, at 37°C with stirring for 6 min. The sample was then placed on ice, followed by addition of EDTA (5 mM final concentration) and 2x protease inhibitor cocktail (Halt^TM^ Protease and Phosphatase Inhibitor, Thermo Scientific, USA) before transfer to 1.5 mL Eppendorf tubes. Platelets and aggregates were removed by serial two-step centrifugation at 1,000 x *g* for 10 min at 4°C, isolating the platelet releasate. Approximately 950 μL releasate was recovered and stored at -80°C. A 60 μL aliquot was reserved for protein analysis (Pierce^TM^ BCA Protein Assay Kit, Thermo Scientific, USA).

### Fractionation and concentration of releasate

Control and thrombin-activated releasate samples were thawed at 37°C, transferred to individual, 3.5 mL, polycarbonate tubes (Beckman Coulter, USA) and centrifuged at 50,000 x *g* for 1 hour at 5°C (OptimaTM L-90K Ultracentrifuge, Beckman Coulter, USA). The supernatants (soluble fraction) and pellets (particulate fraction including microparticles) were separated and the pellets were stored at -80°C. Soluble fractions were concentrated by centrifugal filtration using a 0.5 mL, 15 kDa cut-off filtration device (Merck Millipore, Ireland). A 10 μL aliquot was removed and diluted with 50 μL activation buffer for protein analysis. The remaining concentrate and the flow-through samples were stored at -80°C.

### SDS-page and in gel tryptic digestion

**Fig 2:**
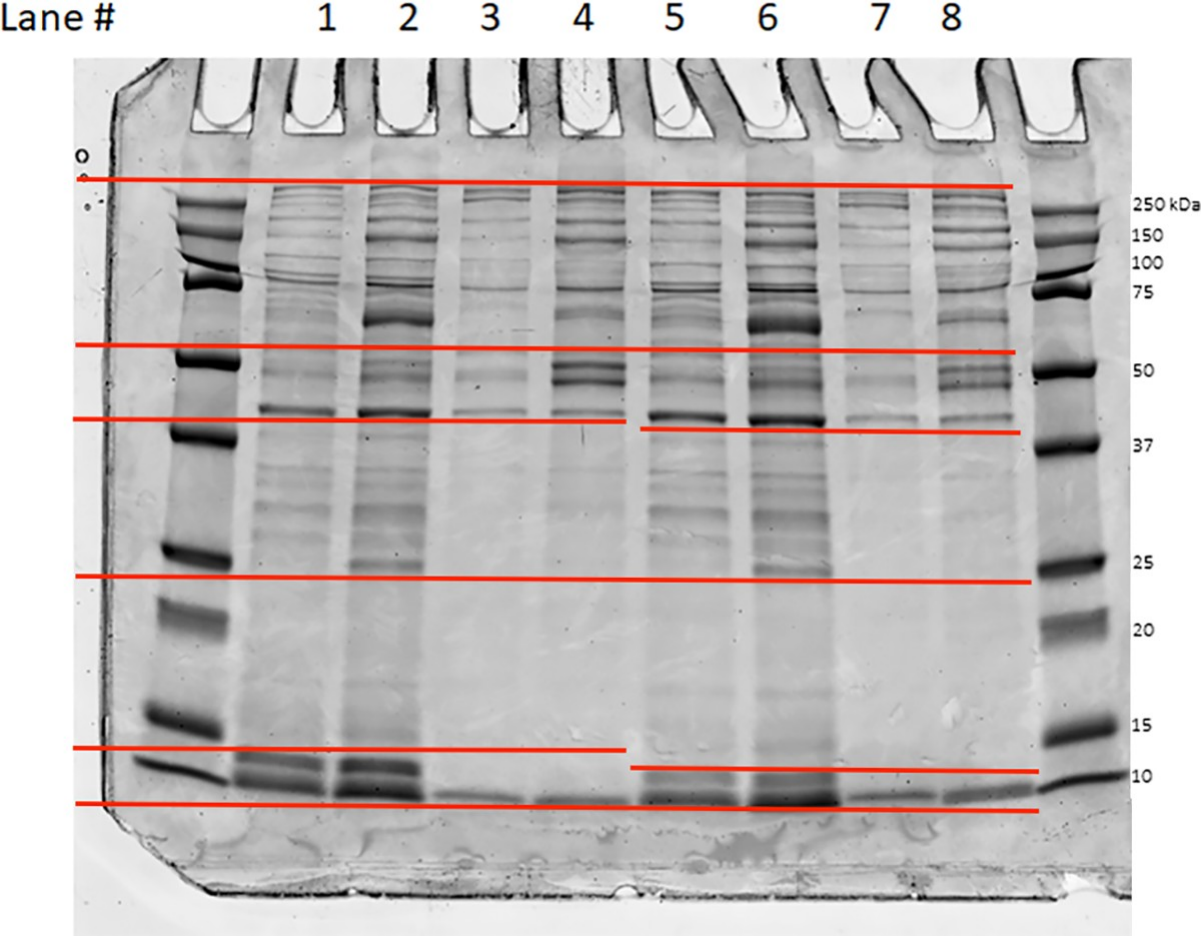
Excision strategy for in-gel digestion. Regions excised for in-gel digestion are shown for two dogs. Soluble fraction control samples (lanes 1 and 5, respectively), soluble fraction thrombin-stimulated samples (lanes 2 and 6, respectively), pellet fraction control samples (lanes 3 and 7, respectively) and pellet fraction thrombin-stimulated samples (lanes 4 and 8, respectively). The red bars indicate the excised regions used to generate tryptic digests for nano-LC and MS/MS. Molecular weight markers are shown.

The four samples generated for each dog (thrombin-stimulated and saline control samples for the soluble and the pellet fractions, respectively) were separated by SDS-page on 12% TGX precast gels (Bio Rad, USA). For the thrombin-stimulated samples, 5 μg was loaded pr. lane, and the equivalent sample volume was loaded for the respective control samples. The gels were fixed in 50% methanol, 43% water, and 7% acetic acid, and then rehydrated prior to colloidal Coomassie blue staining, as previously described (Fig 2) [46]. For each lane of the SDS-PAGE gel, four gel slices equivalent for each dog, were cut based on band intensity into 1 mm cubes and subjected to in-gel digestion followed by extraction of the tryptic peptides as reported previously [53]. The excised gel pieces were washed consecutively in 200 μL distilled water, 100 mM ammonium bicarbonate (Ambic)/ACN (1:1) and ACN. The gel pieces were reduced with 70 μL of 10 mM DTT in 100 mM Ambic for 1 hr at 56°C, alkylated with 100 μL of 55 mM iodoacetamide in 100 mM Ambic at RT in the dark for 60 minutes. After wash steps as described above, the gel slices were dried and rehydrated with 50 μL trypsin in 50 mM Ambic, 10% ACN (20 ng/μL) at 37°C for 16 hrs. The digested peptides were extracted twice with 70 μL of 50% ACN, 5% formic acid (FA) and once with 70 μL of 90% ACN, 5% FA. Extracts from each sample were combined and lyophilized.

### In solution digestion of flow-through samples

Flow-through fractions containing proteins not retained by the filtration device were collected for control and stimulated dog samples. Prior to in-solution proteomic analyses they were pooled for each experimental group. Sample preparation of S-Trap micro spin column (ProtiFi, Huntington, NY, USA) was according to the vendor’s protocol and Zougman *et al*. [54, 55]. Four hundred microliter were taken from each sample. This corresponded to 9.6 μg protein from the stimulated sample and 1.1 μg protein from the control sample, which, respectively, were mixed with 50 mM iodoacetamide (final concentration) for 45 min in the dark at room temperature. 12% aqueous phosphoric acid was added at 1:10 for a final concentration of 1.2%, and S-Trap buffer (90% methanol in 100 mM triethylammonium bicarbonate (TEAB), pH7.1) was added to form colloidal protein particulate. Protein mixtures were transferred into the S-Trap micro column and centrifuged at 4,000 x *g* for 10 min, and washed with 150 μL S-Trap buffer. Finally, 12 μL trypsin buffer (0.5 μg trypsin in 50 mM TEAB) was added and digested at 37°C for 16–18 h. For peptide elution collection, 40 μL TEAB was added into micro spin column and centrifuged at 4,000 x *g* for 10 min and washed twice with 50 μL 50% ACN containing 0.2% FA solution. The digested peptides were dried in Speed Vacuum.

### Nano liquid chromatography and mass spectrometry

Lyophilized tryptic peptides extracted from the gel were reconstituted in a total volume of 73 μL of 0.5% FA for each dog. Reconstitution volumes were varied (10–20 μL) for lyophilized tryptic peptides from gel segments based on their protein content. Peptides were analyzed by nano-LC-ESI MS/MS in single technical replicates [56] using an Orbitrap Fusion^TM^ Tribrid^TM^ (Thermo-Fisher Scientific, San Jose, CA) mass spectrometer equipped with a nanospray Flex Ion Source, and coupled with a Dionex UltiMate 3000 RSLCnano system (Thermo, Sunnyvale, CA) [54, 57]. Reconstituted peptides from individual gel segments and in-solution digests were injected (10–20 μL) onto a PepMap C-18 RP nano trapping column (5 μm, 100 μm i.d. x 20 mm) at 20 μL/min flow rate for rapid sample loading and then separated on a PepMap C-18 RP nano column (2 μm, 75 μm x 25 cm) at 35°C. Column peptide load was monitored by MS signal intensity. The tryptic peptides were eluted in a 120 min gradient of 5% to 38% ACN in 0.1% FA at 300 nL/min, followed by a 7 min ramping to 90% ACN-0.1% FA and an 8 min hold at 90% ACN-0.1% FA. The column was re-equilibrated with 0.1% FA for 25 min prior to the next run. The Orbitrap Fusion was operated in positive ion mode with spray voltage set at 1.6 kV and source temperature at 275°C. External calibration for FT, IT and quadrupole mass analyzers was performed. For in data-dependent acquisition (DDA) analysis, the instrument was operated using FT mass analyzer in MS scan to select precursor ions followed by 3 second “Top Speed” data-dependent collision-induced dissociation ion trap MS/MS scans at 1.6 m/z quadrupole isolation for precursor peptides with multiple charged ions above a threshold ion count of 10,000 and normalized collision energy of 30%. MS survey scanned at a resolving power of 120,000 (FWHM at m/z 200), for the mass range of m/z 375–1575. Dynamic exclusion parameters were set at 40 s of exclusion duration with ±10 ppm exclusion mass width. All data were acquired under Xcalibur 3.0 operation software (Thermo-Fisher Scientific). It should be noted that the nanoLC column used for dog 1 differed from dogs 2 and 3, as dog 1 was analyzed prior to the two other dogs. Stored samples from dog 1 however, were re-analyzed at the same time as dog 2 and 3.

### Protein identification and quantification

The DDA raw files for CID MS/MS were subjected to database searches using Proteome Discoverer 2.2 (PD 2.2) software (Thermo Fisher Scientific, Bremen, Germany) with the Sequest HT algorithm. All four raw MS files of the four gel slices for each soluble and pellet sample (stimulated vs saline control, respectively) were combined for searching against a *Canis lupus familiaris* RefSeq database (CanFam3.1, downloaded from NCBI on Jan. 12, 2018) containing 45,326 entries plus a common contaminant database of 246 entries [58]. Two-missed trypsin cleavage sites were allowed. Peptide precursor tolerance was set to 10 ppm and fragment ion tolerance was set to 0.6 Da. Variable modification of methionine oxidation, deamidation of asparagine/glutamine and fixed modification of cysteine carbamidomethylation were set for the database search. High confidence peptides defined by Sequest HT with a 1% false discovery rate by Percolator were considered for identification. Relative quantitation of identified proteins between thrombin-stimulated and saline control samples, for pellet and soluble fractions, was determined by the Label Free Quantitation workflow in PD 2.2. Precursor abundance intensity for each peptide identified, was automatically determined and unique peptides for each protein were summed and used to calculate the protein abundance (the MS1-abundance) within the PD 2.2 software without normalization. Protein ratios were calculated based on pairwise ratios for stimulated/saline control samples. All protein raw data output files can be found in the supportive information.

### Bioinformatic analysis

Proteins identified in the flow-through samples were combined with those identified in the soluble fractions. Protein lists were curated to remove duplicates and dog RefSeq accession numbers were converted to the homologous human Entrez Gene IDs using bioDBnet (https://biodbnet-abcc.ncifcrf.gov). The proteins were then compared to the reported human global platelet proteome [15], the recently reported core human [29] and murine thrombin releasates[47]. For this, human protein identifiers were mapped to Entrez Gene IDs using the MyGene module in Python (http://www.ebi.ac.uk/Tools/picr/). Canine proteins not matched, were manually reviewed. Proteins were considered a match to a human protein counterpart when identified as a precursor protein, annotated with the qualifier “like protein” or present in a single protein isoform. Redundant protein species were considered splice variants.

Proteins present in a minimum of 2/3 dogs having an MS-1 abundance ratio for paired stimulated/control samples greater than or equal to two [30] were defined as CAPS proteins. This avoided skewing the data set in favor of releasate proteins represented at highest abundance. The CAPS proteins were subjected to Gene Ontology (GO) analysis, and all dog RefSeq gene accession numbers and their GO terms were collected with R Bioconductor (https://bioconductor.org), using R Bioconductor package biomaRt [59]. This set was used for the gene enrichment analysis for gene ontology with R Bioconductor package topGO. Signal pathway enrichment analysis on the Reactome database [60] was performed with Enrichr web server [61]. Lastly, the abundance ranking of the CAPS proteins were manually compared to that of the human [29] and murine [47] secretomes.

## Results

### Sample purity

For all dogs, there was minimal contamination from other cells. No RBC or WBC were detected in CBCs of the final platelet suspensions, and flow cytometry demonstrated <0.1% of CD18-expressing cells (leukocytes). Major RBC proteins, glycophorin A, band 3 anion transporter (both approximately 10^6^ copies /RBC [62]) and hemoglobin (10^9^ copies /RBC [15]) were identified in concentrations indicating a maximum of 1–2 RBC/mL of final platelet suspension (S1 Table). The specific neutrophil protein, CD177 (2.5–7.5 x 10^4^ copies/cell [63]) was found with a maximal MS1 abundance of 1.2 x 10^5^, corresponding to 2–5 neutrophils/mL. The lymphocyte protein, CD83, was present in a single dog in trace amounts.

### Platelet activation status

Post-washing platelet activation status, based on CD62P expression, was low [22] for the three dogs, at 0.7%, 5.9% and 8.1%, respectively. When subjected to gamma-thrombin activation, platelet CD62P expression was 65.1%, 70.6% and 77.9%, respectively. Platelets from all three dogs demonstrated robust aggregation response to thrombin and undetectable aggregation in stirred saline controls (S3 Fig).

### Platelet protein identification

A total of 1,918 platelet proteins were identified from three biological replicates (S1 Table). Individually, 1,090, 1,467 and 1647 proteins were identified from dogs 1, 2 and 3, respectively, and 908 proteins were identified in all three dogs. Of the 1,918 total proteins, 647 were found in the soluble fraction, 447 in the pellet fraction and 824 proteins were found in both fractions. Two or more peptides were used to confidently identify 70.1% of the canine platelet proteins, while 29.9% were identified using a single unique peptide.

**Fig 3:**
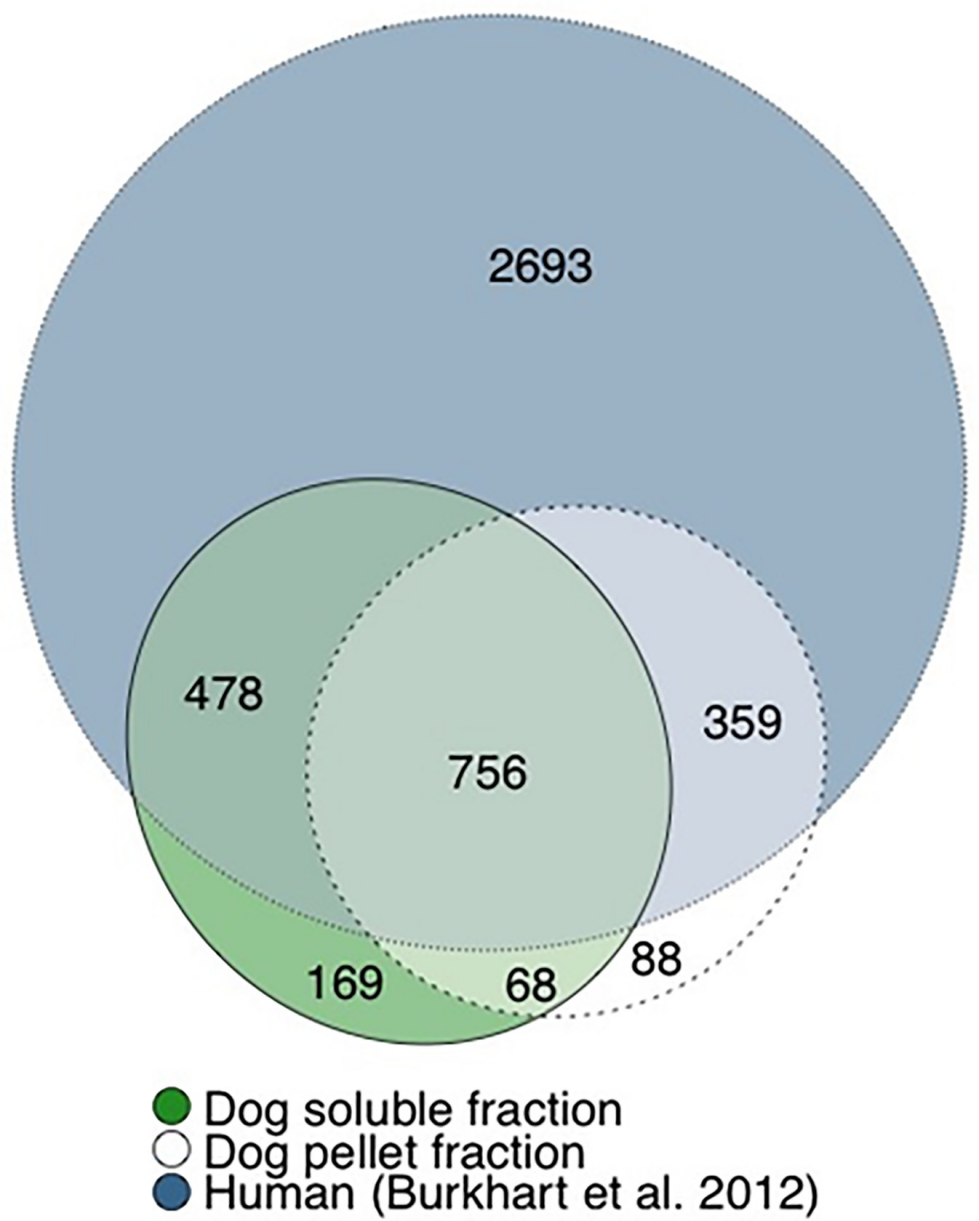
Venn diagram analysis. Comparison of all identified canine platelet proteins to the global platelet lysate proteome reported by Burkhart *et al*. 2012. The diagram shows the number of human lysate proteins shared with the canine secretome (1593), the number of lysate proteins that were not co-identified (2693), and the number of canine proteins that were not reported in the human platelet lysate (325). For the canine proteins, the figure also illustrates the number of proteins in the soluble fraction (647), pellet fraction (447) or both fractions (824).

Of the total canine proteins, 1,593 (83.1%) were also found in the global human platelet proteome of 4,116 proteins reported by Burkhart *et al*. 2012 [15] (S2 Table). Fig 3 shows the number of proteins overlapping between the two datasets, and the fraction (soluble or pellet) in which they were identified. A total of 325 canine proteins were not included in the human global platelet proteome, and of those, 66 (20.3%) were detected in all three dogs. These proteins were searched in PubMed for association with human platelets, megakaryocytes, thrombocytes, alpha-granules and dense granules, and they were searched in a human MS1 proteome database for cell type and tissue expression. Proteins previously associated with platelets or other vascular compartment cells were removed, leaving 3 proteins; C-C motif chemokine 14, teneurin-3 and protein-arginine deiminase type-3, that may represent novel platelet secretion proteins.

### The canine platelet secretome (CAPS)

A total of 693 CAPS proteins were identified (S3 Table), comprising 360 proteins present only in the soluble fraction, 189 only in the pellet fraction, and 144 in both fractions. Fifty CAPS proteins (7.2%) were low abundance, identified from a single unique peptide.

To verify the quality and platelet relevance of the CAPS profile, enrichment analyses of GO-annotations and cell-signal pathways were performed. For biological process, 12 of the top 20 most significantly enriched annotations were directly related to hemostasis, with the top five containing ‘coagulation’, blood coagulation’, ‘hemostasis’, and ‘wound healing’ (Table 1). Significantly enriched GO-annotations for cellular compartment included ‘secretory vesicle’, ‘platelet alpha-granule’, ‘fibrinogen complex’ and ‘secretory granule’ (S4 Table), and molecular function was dominated by ‘binding’ and ‘enzyme regulator activity’ (S5 Table).

**Table 1:** Significantly enriched GO-annotations for ‘biological process’.

GO ID	GO Term	Annotated CAPS proteins	*P*
GO:0050817	Coagulation	30	2.7e-18
GO:0007596	Blood coagulation	29	3.2e-18
GO:0007599	Hemostasis	29	5.7e-18
GO:0052547	Regulation of peptidase activity	43	8.1e-18
GO:0042060	Wound healing	39	3.5e-17
GO:0010951	Negative regulation of endopeptidase activity	32	1.4e-16
GO:0006508	Proteolysis	100	1.6e-16
GO:0052548	Regulation of endopeptidase activity	40	2.4e-16
GO:0010466	Negative regulation of peptidase activity	32	3.9e-16
GO:0009611	Response to wounding	41	6.6e-16
GO:0045861	Negative regulation of proteolysis	36	3.7e-15
GO:0072376	Protein activation cascade	15	9.7e-15
GO:0072378	Blood coagulation, fibrin clot formation	9	4.8e-14
GO:1901564	Organonitrogen compound metabolic process	250	7.5e-14
GO:0030193	Regulation of blood coagulation	18	1.1e-13
GO:1900046	Regulation of hemostasis	18	1.1e-13
GO:0050818	Regulation of coagulation	19	1.4e-13
GO:0030162	Regulation of proteolysis	52	6.3e-13
GO:0042730	Fibrinolysis	11	7.5e-13
GO:0061041	Regulation of wound healing	20	1.7e-11

**Fig 4:**
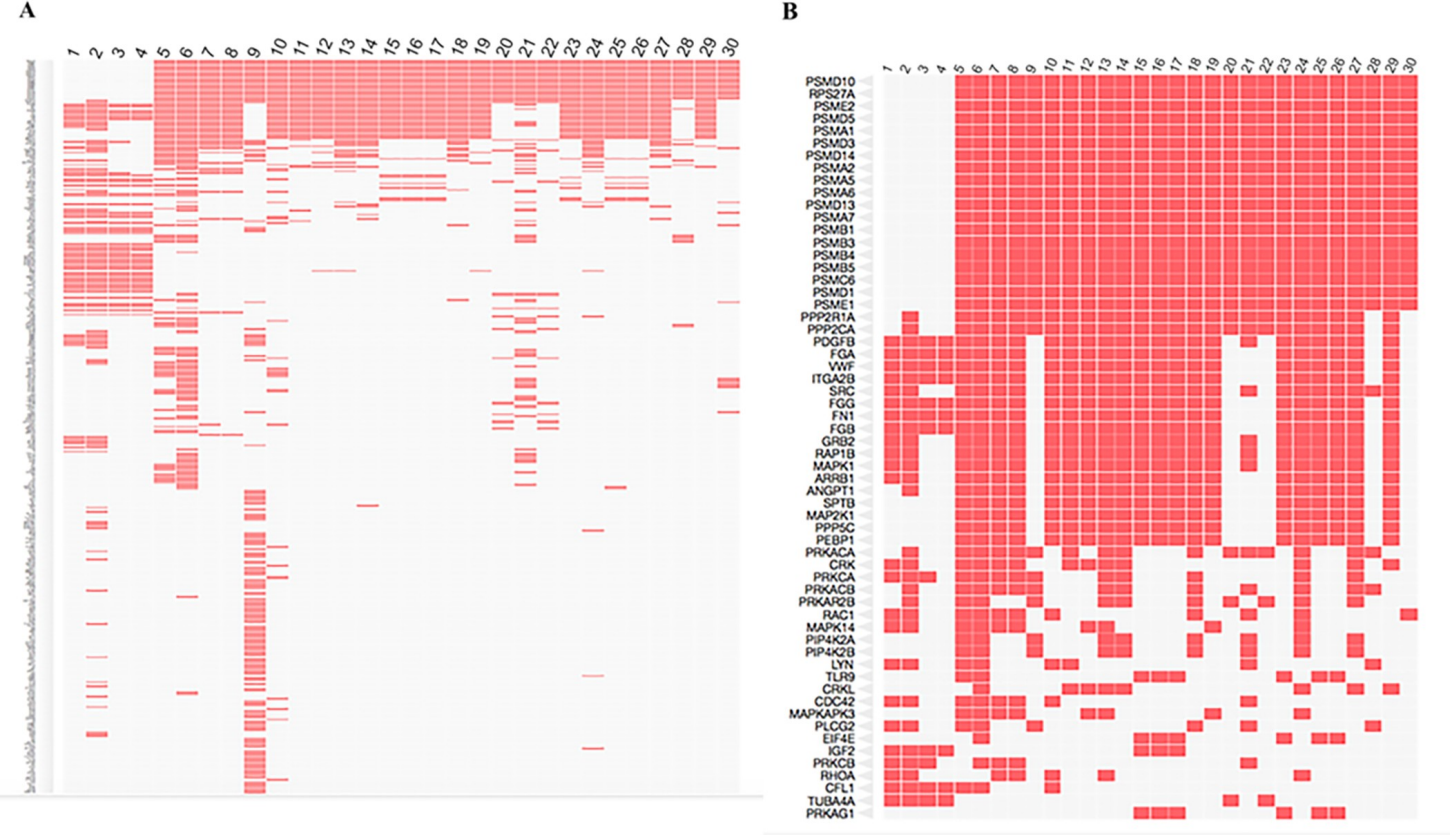
Signal pathway enrichment analysis. **A**: Clustergram illustrating the relationship between CAPS proteins (rows) and the 30 most enriched pathways (columns) for the 693 CAPS proteins. Proteins are ranked within a pathway from high to low frequency, and the pathways* are ranked according to the significance-level of enrichment (decreasing enrichment-level left to right). Proteins rarely enriched and unique to individual pathways cluster near the bottom. **B**: Highlight of the 60 most frequently enriched proteins of the clustergram. Approximately 20 proteins are enriched in the majority of the pathways, but not in the most significantly enriched (pathways 1–4), which share a similar and distinct protein fingerprint. *) Pathways: 1. Platelet activation, signaling and aggregation. 2. Hemostasis. 3. Response to elevated platelet cytosolic Ca^++^. 4. Platelet degranulation. 5. Innate immune system. 6. Immune system. 7. Signaling by VEGF. 8. VEGFA-VEGFR2 pathway. 9. Metabolism. 10. Axon guidance. 11. Interleukin-3, 5 and GM-CSF signaling. 12. Signaling to ERKs. 13. NGF-signaling via TRKA from the plasma membrane. 14. Signaling by PDGF. 15. IGF1R signaling cascade. 16. IRS-related events triggered by IGF1R. 17. Signaling by Type 1 IGF1R. 18. DAP12 signaling. 19. Signaling to RAS. 20. G2/M transition.21. Adaptive Immune System. 22. Mitotic G2-G2/M phases. 23. IRS-mediated signaling. 24. Signaling by NGF. 25. Signaling by NGF receptor. 26. Insulin-receptor signaling cascade. 27. Downstream signal transduction. 28. C-type lectin receptors (CLRs). 29. Prolonged ERK activation events. 30. Host interactions of HIV factors.

Fig 4 shows the association of the CAPS proteins with the 30 most enriched signal pathways. The top 20 proteins of the clustergram were enriched in all of the 30 listed pathways except for the top four pathways; ‘platelet activation, signaling and aggregation’, ‘hemostasis’, ‘response to elevated platelet cytosolic Ca^++^’, and ‘platelet degranulation’. These four pathways had a more distinct and highly similar protein fingerprint.

The table lists the 20 most significantly enriched GO-annotations for the 693 CAPS proteins found in at least 2/3 dogs with a stimulated/control ratio equal or above two.

Analysis link: http://amp.pharm.mssm.edu/Enrichr/enrich?dataset=72rsm

The 693 CAPS proteins were compared to the 276 core human proteins and the top 100 murine proteins identified in the releasate of thrombin-activated platelets reported by Parsons *et al*. 2018 [29] and Martín-Granado *et al*. 2017 [47], respectively (S3 Table). Of the human core, 160 proteins (58%) were co-identified, and another 62 (22.5%) were detected in the subset of canine proteins that had MS1 sample/control ratios less than two (S1 Table). Fifty-four of the human core proteins (19.5%) were not identified in the canine platelet fractions we analyzed (Table 2). Of the murine secretome proteins, 78% were found in the dogs (S1 Table) and 50% were found among the CAPS proteins (S3 Table).

**Table 2:** Human secretome proteins not found in dogs.

Gene name	Protein name
*ACTB*	Actin, cytoplasmic 1
*APLP2*	Amyloid-like protein 2
*APOA2*	Apolipoprotein A-II
*APOA4*	Apolipoprotein A-IV
*APOB*	Apolipoprotein B-100
*APOC1*	Apolipoprotein C-I
*C9*	Complement component C9
*CCL5*	C-C motif chemokine 5
*CD36*	Platelet glycoprotein 4
*CFHR1*	Complement factor H-related protein 1
*CTGF*	Connective tissue growth factor
*CTSW*	Cathepsin W
*CXCL3*	C-X-C motif chemokine 3
*DAG1*	Dystroglycan
*FSTL1*	Follistatin-related protein 1
*GP5*	Platelet glycoprotein V
*HSPA1B*	Heat shock 70 kDa protein 1B
*IGHA1*	Ig alpha-1 chain C region
*IGHG2*	Ig gamma-2 chain C region
*IGHG3*	Ig gamma-3 chain C region
*IGHG4*	Ig gamma-4 chain C region
*IGHM*	Ig mu chain C region
*IGHV3-23*	Ig heavy chain V-III region TIL
*IGHV3-33*	Ig heavy chain V-III region KOL
*IGHV3-72*	Immunoglobulin heavy variable 3–72
*IGKC*	Ig kappa chain C region
*IGKV1-5*	Ig kappa chain V-I region EU
*IGKV2D-28*	Ig kappa chain V-II region TEW
*IGKV3-20*	Ig kappa chain V-III region WOL
*IGKV4-1*	Ig kappa chain V-IV region Len
*IGLC2*	Ig lambda-2 chain C regions
*ITIH3*	Inter-alpha-trypsin inhibitor heavy chain H3
*LGALS3BP*	Galectin-3-binding protein
*MMP1*	Interstitial collagenase
*N/A*	Uncharacterized protein
*NRGN*	Neurogranin
*NUCB1*	Nucleobindin-1
*ORM1*	Alpha-1-acid glycoprotein 1
*ORM2*	Alpha-1-acid glycoprotein 2
*PCSK6*	Proprotein convertase subtilisin/kexin type 6
*PDGFD*	Platelet-derived growth factor D
*PF4*	Platelet factor 4
*PF4V1*	Platelet factor 4 variant
*QSOX1*	Sulfhydryl oxidase 1
*RAB27B*	Ras-related protein Rab-27B
*RNASET2*	Ribonuclease T2
*S100A4*	Protein S100-A4
*SDC4*	Syndecan-4
*SDPR*	Serum deprivation-response protein
*SERPING1*	Plasma protease C1 inhibitor
*SPARC*	SPARC
*ST3GAL6*	Type 2 lactosamine alpha-2,3-sialyltransferase
*TGFBI*	Transforming growth factor-beta-induced protein ig-h3
*VEGFC*	Vascular endothelial growth factor C

The table lists the 54 proteins found in the human core secretome [29] that were not present in the canine dataset.

Quantitative comparisons between the most abundant CAPS and human [29] and murine [47] platelet secretomes are displayed in Table 3 (top 30) and in S6 Table (top 100). Differences included relative deficiencies of canine vWF and coagulation factor V, ranked 288 and 298, respectively (S3 Table), and alpha-2-macroglobulin that did not meet the criteria for a CAPS protein. Among the highly abundant canine releasate proteins (top 20–30), fibronectin, metalloproteinase inhibitor 1 and Trem-like protein 1 were absent in the top 100 of either the human or the murine secretome. Other abundant canine proteins, including the C-C motif chemokine 14 and the potent serine protease inhibitor, protease nexin-1 (PN-1) were absent from the top 100 secreted proteins for both humans and mice.

**Table 3:** Inter-species platelet secretome comparisons.

Canine top 30	Human top 30	Murine top 30
Thrombospondin-1	Serum albumin	Serum albumin
**Fibrinogen alpha chain**	Thrombospondin-1	Thrombospondin 1
**Fibrinogen beta chain**	Platelet basic protein	Serotransferrin
**Fibrinogen gamma chain isoform X1**	**Talin-1**	**Platelet factor 4**
Fibronectin isoform X7	Complement C3	**Alpha-2-macroglobulin**
Serum albumin precursor	Filamin-A	Actin, cytoplasmic 1
Gelsolin	Serotransferrin	**Talin-1**
Platelet basic protein precursor	Myosin-9	Actin, cytoplasmic 2
**C-C motif chemokine 14**	Actin, cytoplasmic 2	**Apolipoprotein A-I**
Profilin-1	**von Willebrand factor**	Hemoglobin subunit alpha
Metalloproteinase inhibitor 1 isoform X1	Multimerin-1	Fibrinogen alpha chain
Tubulin beta-4B chain isoform X2	**Alpha-2-macroglobulin**	Hemoglobin subunit beta-1
Cofilin-1	**Platelet factor 4 variant**	Ig mu chain C region (Fragment)
Tubulin alpha-4A chain isoform X1	Uncharacterized protein	Hemopexin
Peptidyl-prolyl cis-trans isomerase A	Alpha-1-antitrypsin	Beta-2-glycoprotein 1
**Trem-like transcript 1 protein isoform X1**	Gelsolin	**von Willebrand factor**
Tubulin beta-1 chain isoform X1	**Coagulation factor V**	Complement C3
Pleckstrin	Alpha-actinin-1	Serine protease inhibitor A3K
Phosphoglycerate kinase 1	Fibrinogen alpha chain	Plasminogen
Nidogen-1	Latent-transforming GF beta-binding protein 1	Vitamin D-binding protein
Alpha-enolase	**Apolipoprotein A-I**	Beta-actin-like protein 2
**Glia-derived nexin**	Apolipoprotein B-100	Myosin-9
Fermitin family homolog 3	Haptoglobin	Platelet basic protein
Serotransferrin	Amyloid beta A4 protein	Fibrinogen beta chain
Actin, alpha cardiac muscle 1	Fibrinogen beta chain	Carboxylesterase 1C
**Plasminogen activator inhibitor 1 precursor**	Tropomyosin alpha-4 chain	Filamin, alpha (Fragment)
Adenylyl cyclase-associated protein 1 isoform X2	Complement C4-A	Alpha-enolase
Transgelin-2	Vinculin	Fructose-bisphosphate aldolase A
Coagulation factor XIII A chain	Platelet glycoprotein V	Lysozyme C-2
Glyceraldehyde-3-phosphate dehydrogenase	Fibrinogen gamma chain	Murinoglobulin-1

In the table, the top 30 most abundantly secreted platelet proteins in dogs are compared with those of humans [29] and mice [47]. Noteworthy differences are highlighted in bold.

## Discussion

The present study is the first proteomic profiling to define a canine secretome, or CAPS, representing platelet response to thrombin stimulation. The reactome derived from GO annotation confirms the role of the CAPS proteins in platelet activation and demonstrates conservation across species in platelet participation in the immune response and platelet metabolism. The observed clustering of CAPS proteins within functional pathways provides a resource to explore the contribution of individual platelet proteins in the complex processes of hemostasis, immune response, and tissue repair.

We obtained a highly pure platelet suspension, and documented platelet activation status, including aggregability, in washed, unstimulated control samples and after thrombin stimulation. In the characterization of CAPS proteins, we considered the background signal from unstimulated negative controls to account for the presence of platelet proteins likely derived from *in-vitro* platelet manipulations and not representative of the activation response specifically induced by gamma-thrombin. Our study identified 1,918 total canine platelet proteins and of those, characterized the thrombin-activated CAPS as a subset consisting of 693 proteins. In comparison, three previous studies of thrombin receptor (PAR-receptor) activated human platelets identified between 315–894 proteins in total [24, 25, 29]. These studies and other qualitative descriptions of major human alpha-granule proteins included many CAPS proteins such as platelet basic protein, thrombospondin, vWF, serglycin, vitronectin, fibrinogen, factor V, vitamin K-dependent protein S, plasminogen, platelet-derived growth factor and plasminogen activator inhibitor 1. Similarly, both human and canine secretomes included lysosomal proteins such as cathepsins and heparanase and major membrane proteins like GPIIb-IIIa, GPIb-IX, GPVI, P-selectin, and CD9. As in a recent human report [37], the CAPS proteins in soluble and pellet platelet fractions overlapped. This co-localization of soluble and particulate proteins may represent binding or adsorption of soluble proteins to the particulate fraction (e.g. fibrinogen, vWF, factor V, complement factors), or aggregates of soluble protein complexes (e.g. thrombospondin-fibrinogen or immunoglobulins complexes). De Palio *et al*. 2018 demonstrated the complex composition of the extracellular vesiculosome, containing multivesicular alpha-granules and cytoplasmic vacuoles containing organelles, e.g. mitochondria [35].

In addition to many similarities between dogs and humans, we also found several interspecies differences, including a subset of proteins that can be grouped into chemokines/cytokines, immunoglobulins, growth factors and proteins involved in lipid metabolism (Table 2). We moreover found some unexpected differences between CAPS proteins and the human platelet secretome. The absence of platelet factor 4 in dogs is a novel finding. Platelet factor 4 and beta-thromboglobulin are cleavage products of platelet basic protein found in high abundance in human platelet secretomes. These products were absent from the CAPS and neither protein has a canine entry in the Uniprot database. A basic local alignment (BLAST) of human platelet factor 4 with canine proteins in the NCBI database identified 3 homologous proteins: platelet basic protein precursor, chemokine C-X-C motif ligand 7, and interleukin-8 (IL-8). All of these were defined as CAPS proteins suggesting that, in the dog, platelet basic protein is not cleaved into platelet factor 4 or beta-thromboglobulin, but rather is cleaved into other chemokines and cytokines of similar function, not found in the human secretome [29]. Our finding of high canine platelet IL-8 content, compared to humans, supports a previous report of strong correlation of IL-8 with platelet count in dogs [64].

Using mass spectrometry detection, we also identified three proteins that are uniquely present in canine platelets: C-C motif chemokine 14, teneurin-3 and protein-arginine deiminase type-3. Of these, C-C motif chemokine 14 was found to be a highly abundant CAPS protein. After proteolytic activation, C-C motif chemokine 14 acts as a strong agonist on chemokine receptors (CCR1 and CCR5), with downstream calcium mobilization, and resultant chemotaxis of monocytes, macrophages, T-lymphocytes and eosinophils [65]. The pro-fibrinolytic proteases, urokinase-type and tissue-type plasminogen activators (uPA and tPA), are strong activators of C-C motif chemokine 14 [65], and the active chemokine has also been found to mediate anti-viral effects through CCR5 internalization, a major entry protein for human immunodeficiency virus [66]. In humans, this chemokine is a high concentration plasma protein [67], and potential contamination of the canine platelet secretome with plasma-derived proteins cannot be ruled out. However, it is less probable for proteins with high activated/control MS1 ratios like C-C motif chemokine 14.

Species-differences were also apparent in the relative abundance of releasate proteins. PN-1 was a highly abundant CAPS protein with low or undetectable abundance in the human and murine secretomes, respectively. PN-1, also known as SERPINE2 and glia derived nexin, has been identified as an alpha-granule protein [68] with very low plasma concentration [69]. As a potent thrombin inhibitor, has strong antithrombotic properties [68], as well as anti-fibrinolytic effects, through inhibition of uPA, tPA and plasmin [70]. Fibronectin, metalloproteinase inhibitor-1 and Trem-like 1 also had significantly lower estimated abundances in human and murine releasates vs. CAPS. In contrast, the CAPS proteins were relatively deficient in other proteins. For example, vWF ranked 16 and 28 in abundance for murine [47] and human [29] platelets, respectively, but in the canine platelet secretome, it ranked 288. Together, these features of CAPS render the dog a potential model for defining the actions of proteins unique to CAPS such as C-C motif chemokine 14 and PN-1, or for studying proteins relatively deficient in canine platelet, e.g. the contribution of platelet vs. non-platelet vWF pool.

Our study examined the response to thrombin, a physiologic platelet agonist, and identified CAPS proteins based on comparisons with paired unstimulated control samples. While this allowed us to account for potential non-specific *in-vitro* platelet activation, and more accurately define CAPS in the controlled agonist-induced releasate, it complicates direct comparisons with previous studies. Secretomes reported by others do not include negative (unstimulated) controls, and could include a contaminating cohort of non-secreted platelet proteins. The negative control samples of the present study were processed identically to the activated samples, with substitution of an equivalent volume of saline for thrombin. No subsequent steps were included to prevent potential platelet activation during releasate collection in either saline controls or thrombin-stimulated samples. While unlikely to have influenced core protein profiles for moderate or low abundance proteins; the observed control/stimulated ratio differences for high abundance core proteins may have been somewhat reduced. Additional method-related differences from previous human reports include the use of human gamma-thrombin. This hydrolyzed form of gamma-thrombin selectively activates platelet protease-activated receptor (PAR) 4 [71], and importantly lacks the ability to interact with fibrinogen to form a fibrin clot [72]. This is a major advantage for isolation of secreted proteins, as the presence of an unwanted fibrin clot may influence the proteomic signature for the secreted proteins, through removal of proteins adsorbed to fibrin, platelet aggregates and cell debris. This advantage was also reflected in the canine dataset by significantly lower abundance for cytoskeletal-associated proteins, e.g. talin-1. The overall CAPs protein profile may also have been influenced by method of protein extraction, solubilization and purification, and their possible association with platelet aggregates and debris, removed during sample fractionation. The generation of tryptic peptides in-gel vs in-solution can moreover influence the proteins detected, including basic and hydrophobic proteins, with higher recovery of less abundant proteins favored by use of in-gel digestion [73].

The differing experimental conditions between the present study and those of the human and murine secretomes limit the comparison of the secretome protein profiles. Methods used for proteomics continue to evolve with advances in technology and are a potential source of variation between protein profiles for canine, human and murine secretomes. Another potential limitation of our study was that platelet suspensions used for activation were not adjusted to a standard platelet concentration, and the variable platelet recoveries could reflect differences in baseline platelet numbers, platelet loss during washing, or platelet activation. We compensated for this through standardized sample loading during MS analysis. The very high number of proteins identified in our study compared to previous reports indicates that our protocol was capable of detecting even trace secretome proteins. Differences in the number of proteins identified between dogs may also reflect potential analytical variation for the nanoLC-MS/MS, which may account for the significantly lower total number of proteins identified in dog 1. For this reason, we established presence in 2/3 (not 3/3) dogs as a criterion for identification of CAPS proteins. As for many human studies, this small number of dogs represents a starting point for the method development and preliminary characterization of CAPS. The extent of inter-individual biological variation in the platelet releasate among dogs remains to be established using a larger sample size, as recently described for humans [29].

## Conclusions

We have described a detailed and highly sensitive protocol for identification of proteins secreted from thrombin-activated canine platelets. Using this method, we have taken the first step in defining a reference database for healthy CAPS proteins. Similarities between CAPS and the human secretome confirm the utility of dogs as translational models of human disease. But our dataset also allowed identification of important species differences and findings potentially unique to dogs. Among these differences were replacement of platelet factor 4 with alternative cleavage products of platelet basic protein, novel and/or highly secreted platelet proteins like C-C motif chemokine 14 and PN-1, and markedly lower abundance of functionally significant human alpha-granule proteins, like vWF. These results provide a platform for further investigations into disease-related CAPS profiles and for future comparative proteomic studies to unravel the role of platelet activation in acquired disease shared by dogs and humans, e.g. cancer.

## Supporting information

S1 Fig Flow cytometric gating strategy. Gating strategy for the flow cytometric analysis of platelet CD62P expression. Platelets were sequentially identified by means of forward scatter, side scatter and CD9 expression (top and middle panels). To assess post-wash platelet activation and platelet responsiveness, CD62P (P-selectin) expression of the CD9-positive platelet population (bottom panel), was analyzed relative to isotype control (red population), in the post-wash platelet samples before (light blue population) and after (green population) gamma-thrombin stimulation. FSC: forward scatter, SSC: side scatter.
(TIF)

S2 Fig Flow cytometric gating strategy. Gating strategy for leukocyte identification by means of CD18 expression. Gates for granulocytes and monocytes/lymphocytes were identified using lysed canine whole blood (top panel). CD18 expression relative to isotype control (red population), were documented for granulocytes (middle panel, light blue population) and for monocytes/lymphocytes (bottom panel, blue population). The template was subsequently applied in the assessment of leukocyte contamination of the washed platelets samples. FSC: forward scatter, SSC: side scatter.
(TIF)

S3 Fig Light transmittance aggregometry. Representative aggregation traces for washed platelets following addition of 50 nM gamma-thrombin (trace 1–4) or 0.15 M NaCl_2_ vehicle control (trace 5–8). Increasing light-transmission shown by arrow.
(TIF)

S1 Table Mother table for all proteins identified. The listed 1,918 proteins are sorted A-Z. 1 peptide ID (marked in red): n = 573 (29.9%). 'ID only' designates proteins present in trace amounts, a blank space means the protein was not detected.
(XLSX)

S2 Table Canine proteins also reported in the global platelet proteome. The 1,593 proteins found in both the dogs and in the Burkhart et al. 2012 global platelet proteome. The proteins are listed A-Z. Splice variants are in italic and 1 peptide identifications (n = 392, 24.6%) are marked in red.
(XLSX)

S3 Table CAPS proteins. The 693 proteins found in at least 2/3 dogs with a stim/control ratio ≥ 2. The table is divided into proteins found in both the soluble and the pellet fraction (n = 144), proteins solely found in the soluble fraction (n = 360), and proteins solely found in the pellet fraction (n = 189). They are further sorted based on highest to lowest MS1-abundance. Fifty proteins (7.2%) total were identified with a single peptide.
(XLSX)

S4 Table Significantly enriched GO-annotations for ‘cellular compartment’. GO-annotations that were significantly enriched for the 693 CAPS proteins found in at least 2/3 dogs with a stim/control ratio ≥ 2.
(XLSX)

S5 Table Significantly enriched GO-annotations for ‘molecular function’. GO-annotations that were significantly enriched for the 693 CAPS proteins found in at least 2/3 dogs with a stim/control ratio ≥ 2.
(XLSX)

S6 Table Inter-species platelet secretome comparisons. The top 100 most abundantly secreted platelet proteins in dogs are compared with those of humans [29] and mice [47]. Noteworthy differences are highlighted in bold.
(XLSX)

S7 Table Protein raw data files. All protein raw data files generated from Dog 1 (D1).
(XLSX)

S8 Table Protein raw data files. All protein raw data files generated from Dog 2 (D2).
(XLSX)

S9 Table Protein raw data files. All protein raw data files generated from Dog 3 (D3).
(XLSX)

S10 Table Protein raw data files. All protein raw data files generated for pooled flow-through samples from Dog 1–3.
(XLSX)
