# Supplementary figures and images for: Proteomic profiling of the thrombin-activated canine platelet secretome (CAPS)
Source: PLoS One. 2019 Nov 13;14(11):e0224891. doi: 10.1371/journal.pone.0224891 (PMC6853320; doi:10.1371/journal.pone.0224891)

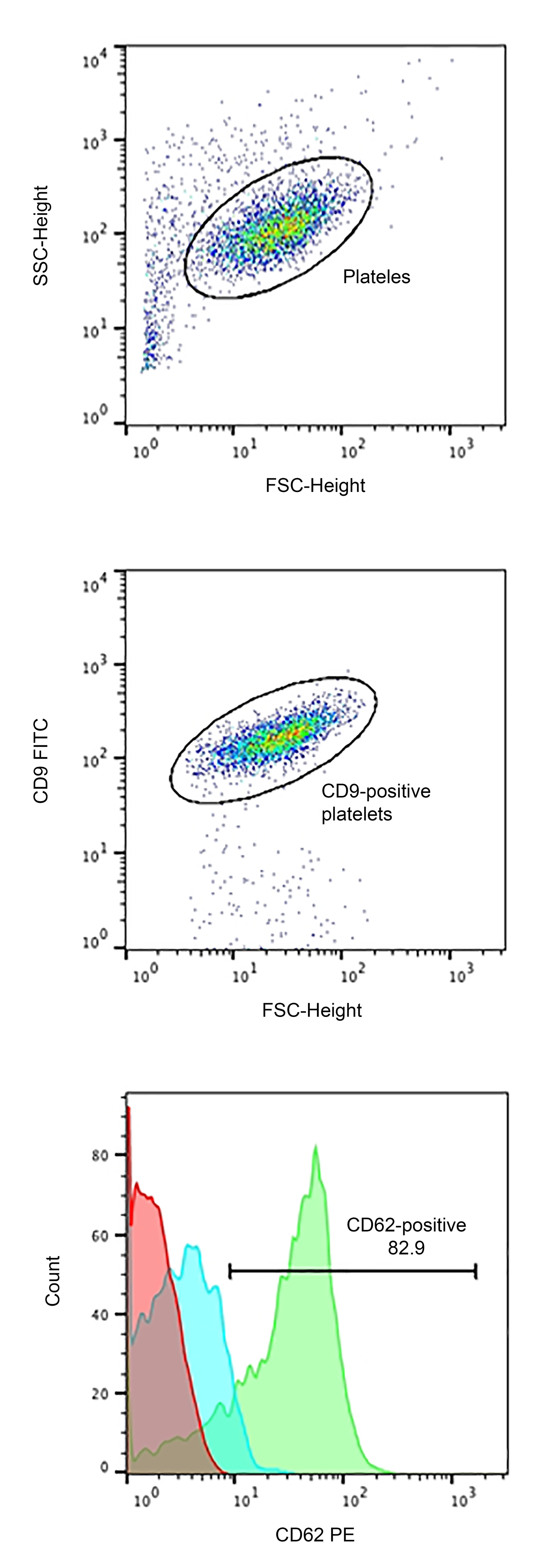

Supplement: S1 Fig — Gating strategy for the flow cytometric analysis of platelet CD62P expression. Platelets were sequentially identified by means of forward scatter, side scatter and CD9 expression (top and middle panels). To assess post-wash platelet activation and platelet responsiveness, CD62P (P-selectin) expression of the CD9-positive platelet population (bottom panel), was analyzed relative to isotype control (red population), in the post-wash platelet samples before (light blue population) and after (green population) gamma-thrombin stimulation. FSC: forward scatter, SSC: side scatter. (TIF) [file pone.0224891.s001.tif]

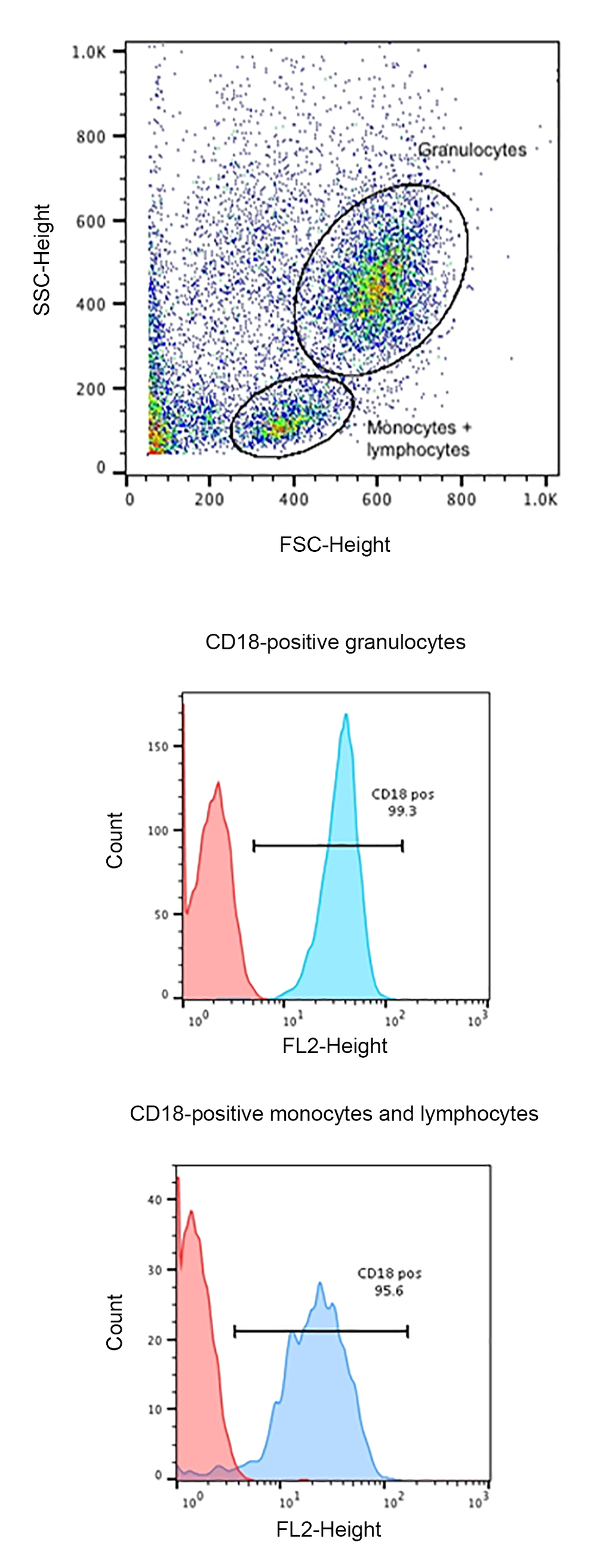

Supplement: S2 Fig — Gating strategy for leukocyte identification by means of CD18 expression. Gates for granulocytes and monocytes/lymphocytes were identified using lysed canine whole blood (top panel). CD18 expression relative to isotype control (red population), were documented for granulocytes (middle panel, light blue population) and for monocytes/lymphocytes (bottom panel, blue population). The template was subsequently applied in the assessment of leukocyte contamination of the washed platelets samples. FSC: forward scatter, SSC: side scatter. (TIF) [file pone.0224891.s002.tif]

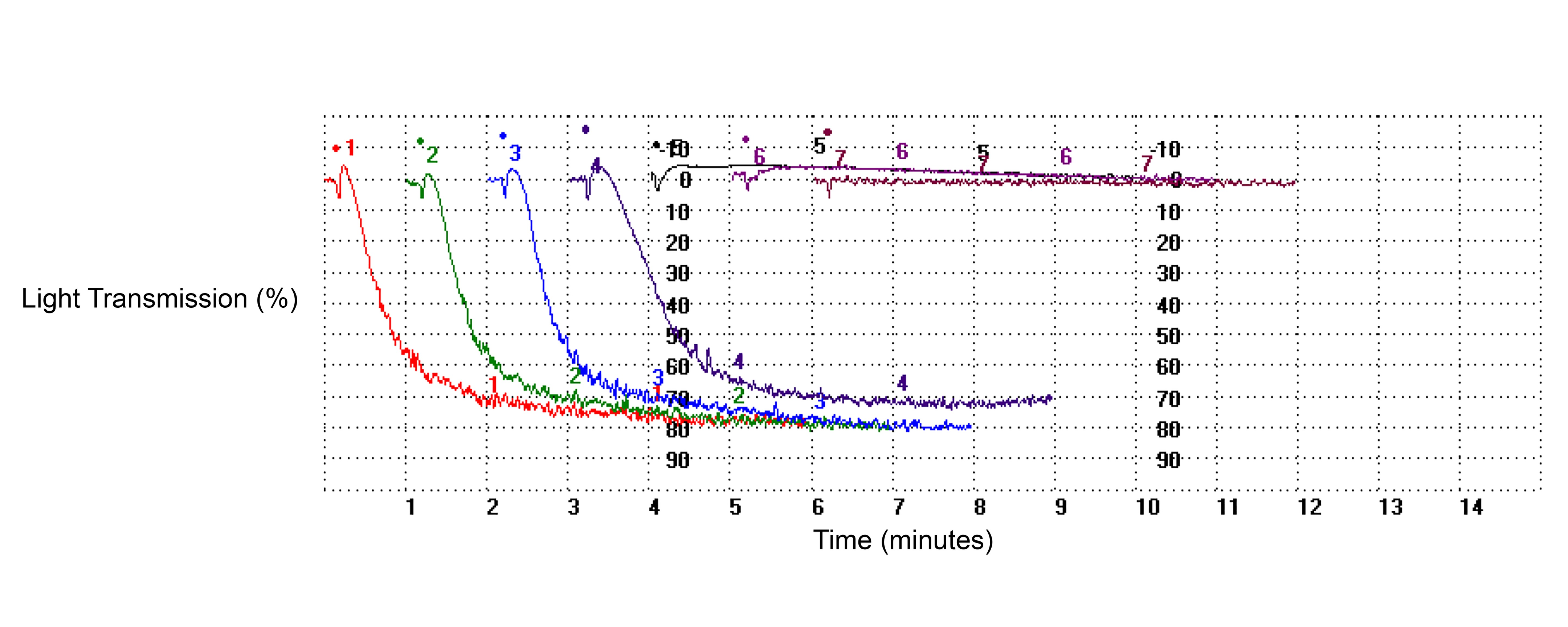

Supplement: S3 Fig — Representative aggregation traces for washed platelets following addition of 50 nM gamma-thrombin (trace 1–4) or 0.15 M NaCl2 vehicle control (trace 5–8). Increasing light-transmission shown by arrow. (TIF) [file pone.0224891.s003.tif]
